# The role of super-spreading events in *Mycobacterium tuberculosis* transmission: evidence from contact tracing

**DOI:** 10.1186/s12879-019-3870-1

**Published:** 2019-03-12

**Authors:** Yayehirad A. Melsew, Manoj Gambhir, Allen C. Cheng, Emma S. McBryde, Justin T. Denholm, Ee Laine Tay, James M. Trauer

**Affiliations:** 1 Department of Epidemiology and Preventive Medicine, School of Public Health and Preventive Medicine, Monash University, Melbourne, Victoria Australia; 2 Department of Epidemiology and Biostatistics, Institute of Public Health, University of Gondar, Gondar, Ethiopia; 3 Australian Institute of Tropical Health and Medicine, James Cook University, Townsville, Queensland Australia; 4 Department of Medicine at Royal Melbourne Hospital, University of Melbourne, Melbourne, Victoria Australia; 5 The Victorian Tuberculosis Program at the Peter Doherty Institute, Melbourne, Victoria Australia; 6 Department of Microbiology and Immunology, University of Melbourne, Melbourne, Victoria Australia; 7 Department of Health and Human Services, Health Protection branch, Melbourne, Victoria Australia

**Keywords:** Tuberculosis, Super-spreading, Negative binomial distribution, Victoria

## Abstract

**Background:**

In current epidemiology of tuberculosis (TB), heterogeneity in infectiousness among TB patients is a challenge, which is not well studied. We aimed to quantify this heterogeneity and the presence of “super-spreading” events that can assist in designing optimal public health interventions.

**Methods:**

TB epidemiologic investigation data notified between 1 January 2005 and 31 December 2015 from Victoria, Australia were used to quantify TB patients’ heterogeneity in infectiousness and super-spreading events. We fitted a negative binomial offspring distribution (NBD) for the number of secondary infections and secondary active TB disease each TB patient produced. The dispersion parameter, *k*, of the NBD measures the level of heterogeneity, where low values of *k* (e.g. *k < 1*) indicate over-dispersion. Super-spreading was defined as patients causing as many or more secondary infections as the 99th centile of an equivalent homogeneous distribution. Contact infection was determined based on a tuberculin skin test (TST) result of ≥10 mm. A NBD model was fitted to identify index characteristics that were associated with the number of contacts infected and risk ratios (RRs) were used to quantify the strength of this association.

**Results:**

There were 4190 (2312 pulmonary and 1878 extrapulmonary) index TB patients and 18,030 contacts. A total of 15,522 contacts were tested with TST, of whom 3213 had a result of ≥10 mm. The dispersion parameter, *k* for secondary infections was estimated at 0.16 (95%CI 0.14–0.17) and there were 414 (9.9%) super-spreading events. From the 3213 secondary infections, 2415 (75.2%) were due to super-spreading events. There were 226 contacts who developed active TB disease and a higher level of heterogeneity was found for this outcome than for secondary infection, with *k* estimated at 0.036 (95%CI 0.025–0.046). In regression analyses, we found that infectiousness was greater among index patients found by clinical presentation and those with bacteriological confirmation.

**Conclusion:**

TB transmission is highly over dispersed and super-spreading events are responsible for a substantial majority of secondary infections. Heterogeneity of transmission and super-spreading are critical issues to consider in the design of interventions and models of TB transmission dynamics.

**Electronic supplementary material:**

The online version of this article (10.1186/s12879-019-3870-1) contains supplementary material, which is available to authorized users.

## Background

The Global Tuberculosis (TB) Strategy looks towards the ultimate vision of elimination of the TB epidemic, although the disease still causes more than 10 million cases and 1.8 million deaths each year [1, 2]. The epidemic is not homogeneously distributed, but is a collection of heterogeneous local micro-epidemics [3]. The existence of heterogeneity in transmission has the potential to disrupt elimination strategies, many of which assume broadly similar transmission potential of infectious people. Hence, it is essential to understand and quantify the degree of heterogeneity that exists in TB transmission.

Several studies have reported heterogeneity in the capacity of individual source patients to transmit various pathogens to their contacts [4–7]. Variation between infectious individuals in their capacity to transmit infectious agents is well described, with some super-spreaders infecting large number of contacts while others may only infect very few or none [4, 5, 7–10]. The contribution of super-spreading has previously been quantified for directly transmitted infections such as SARS, measles, smallpox, monkey-pox and pneumonic plague [4].

TB patients are diverse in their capacity to transmit infection to their contacts, with a systematic review that included several contact tracing studies showing that clinical, demographic and behavioural characteristics of TB patients were associated with their ability to transmit *Mycobacterium tuberculosis* (*M. tb*) infection [11]. Quantification of heterogeneity in *M. tb* transmission will help to understand better how its transmission is sustained. A study in the Netherlands using genotypic clustering data quantified *M. tb* transmission heterogeneity and reported signs of super-spreading [10]. However, how TB patients vary with respect to their capacity to produce secondary infection is not well understood, including the extent to which super-spreading events exist and are responsible for driving transmission. Understanding transmission heterogeneity and characterising those with greater capacity to spread the infection is critical to better target interventions and predict their likely impact. As the global TB response transitions towards ending TB and the epidemic becomes more localised [3], understanding heterogeneities of transmission is increasingly important. We aimed to characterise transmission heterogeneity in a well-resourced setting using high-quality surveillance data including detailed information on contacts and their infection status.

## Methods

### Setting

Victoria is a state of Australia with approximately 5.6 million people and a single centralised tuberculosis program (the Victorian Tuberculosis Program; VTP). Notification of all confirmed or suspected cases of TB disease is mandatory for both laboratories and clinicians and culture confirmation of *M. tb* is routine in this setting. While hospitalisation of cases is not mandatory, those with pulmonary disease are typically maintained in isolation until they are considered non-infectious (> 2 weeks of effective therapy and/or smear-negativity) [12, 13]. On receipt of a notification, a public health nurse from the VTP is allocated to the patient to provide support, assist with treatment compliance, and assess the extent of contact tracing required. Household contacts and others with greater than an estimated 8 h of contact are considered eligible for screening, with individualised assessment of screening recommendations for higher-risk contacts performed (e.g. immunosuppressed or those with high-intensity exposure).

Contact investigation initially consists of clinical assessment and serial testing for *M. tb* infection. Testing of contacts is conducted by either tuberculin skin testing (TST) using the Mantoux procedure or an interferon gamma release assay (IGRA), although during the study period the large majority of testing was undertaken with TST. Those negative on initial testing are tested again 8–12 weeks following exposure. Contacts with either symptoms suggestive of active disease or a positive test for TB infection undergo chest x-ray (CXR) and further clinical assessment, with isoniazid preventive treatment offered for those where active disease is excluded [12].

### Data

Data from the VTP which are stored by the Victorian Department of Health and Human Services (DHHS) were used for the following analysis. Index patients were classified as confirmed cases of TB notified from 1 January 2005 to 31 December 2015 in residents of Victoria. The data set includes contact tracing information and results of testing for *M. tb* infection, with cases of subsequent active TB disease linked to these contact episodes now extending to March 2017 (see [14] for earlier publication of linkage process). We constructed empirical offspring distributions from the detailed contact tracing data set of the VTP.

Ethical approval was obtained from Monash University, Human Research Ethics Committee (Project Number: 7776) and permission was given by the VTP and DHHS.

### Definitions

A microbiologically confirmed case of TB requires culture or polymerase chain reaction (PCR) diagnosis of *Mycobacterium tuberculosis*, while clinical/radiological diagnosis may also be made by a medical practitioner experienced in TB management. Approximately 90% of TB cases in Victoria are bacteriologically confirmed [15]. All cases diagnosed with active TB in this dataset also underwent secondary case review by a TB specialist to ensure that guidelines for confirming TB disease were met.

Contacts were individuals identified as having had personal contact with index patients by the VTP, through school, workplace, household and other settings. Contact latent TB infection (LTBI) was defined as a TST result of ≥10 mm in an identified contact. Where contacts had had multiple TSTs, we used the value of the latest TST result performed within three months of exposure. Any contact developing active TB during the stated period until 21 March 2017 was considered to have secondary TB. We define a super-spreading event as the number of secondary infections per index case that was greater than the 99th centile of the equivalent Poisson distribution (with distribution mean equal to the mean number of infections per index).

### Fitting distributions to data

We were primarily interested in the distribution of the number of secondarily infected contacts from each index patient. Although super-spreading is usually defined in terms of the number of secondary cases of active disease produced by each index patient, we wished to estimate parameters in the absence of preventive therapy. As isoniazid prophylaxis is used widely in our setting and its use may be clustered according to index patients (e.g. family members electing together to undertake preventive treatment), we anticipated this could artefactually inflate our estimates of over-dispersion. Although there are contact factors that are likely to affect progression to active disease after infection, these factors may not be differentially distributed by index patient and the distribution of infections would be unaffected by use of preventive therapy. Therefore, the number of secondarily infected cases is our primary analysis throughout the remainder of the paper, although analogous analyses are presented for the distribution of contacts (close contacts and all contacts) and secondary cases of active disease to facilitate epidemiological interpretation.

Our primary outcome can be described using a probability distribution termed an offspring distribution, defined as the probability of the number of transmission events across the range of index TB patients. This process can be modelled by a negative binomial distribution (NBD), which has the advantage of being able to accommodate over-dispersed count data [16–18]. The NBD permits sufficient flexibility with only two parameters (the shape parameter and the mean) [4] and subsumes the Poisson distribution while also allowing for “over-dispersion”, where the variance (of the offspring distribution) may be greater than the mean [18].

We denote the individual reproductive number by *v* and, the distribution of individual reproductive numbers (offspring distribution) by *Z*. To incorporate individual infectious histories, *v* follows a negative binomial offspring distribution with dispersion parameter *k* and mean *m,* such that *Z*~*NegB*(*m*, *k*)*.* The dispersion parameter *k* quantifies the extent of over-dispersion in the count data. If there is extra-heterogeneity between index patients in the number of secondary infections produced, dispersion increases and the parameter *k* approaches zero (k → 0). In the absence of over-dispersion, k → ∞ and the mean and the variance approach parity, with the negative binomial distribution reducing to the Poisson distribution. If *k* = 1, the negative binomial distribution reduces to the geometric distribution, such that the negative binomial model can accommodate Poisson, geometric and over-dispersed distributions [16].

The probability of observing index patients with *v* number of infected contacts is given by:

1 $$ \mathrm{P}\left(Z=v\right)=\frac{\left(k+v-1\right)!}{v!\left(k-1\right)!}.{\left(\frac{m}{m+k}\right)}^v{\left(1+\frac{m}{k}\right)}^{-k},m>0,k>0 $$

As the variance m(1 + (m/k)) approaches the mean (*m*), over-dispersion decreases, i.e. k → ∞.

### Interpretation of transmission parameters

The parameters, *k* and *m* were estimated by maximum likelihood estimation (MLE), which provides unbiased estimates, especially for large sample sizes [16]. The MLE of the mean of the offspring distribution, *m* is the sample mean of *Z* or the mean number of secondary infections. The dispersion parameter, *k*, was estimated after fitting the data to the negative binomial distribution using the MASS package [19] of the R environment for statistical computing [20], with a value of *k* less than one interpreted as evidence of super-spreading [10].

### Super-spreading events

We used the protocol proposed by Lloyd-Smith et al [4] in which: first, we calculated the mean number of secondary infections per index or effective reproductive number, *R*_*n*_; second, we constructed a Poisson distribution with mean *R*_*n*_, representing the range of *Z* (offspring distribution) due to stochasticity without individual variation; third, we define a super-spreading event as any patient who infected more than *Z*(*i*) contacts, where *Z*(*i*) is *i*^*th*^ centile of the offspring Poisson distribution. Arbitrarily but as in this previous study for SARS, we considered the 99th percentile of this distribution as the cut-point to determine super-spreading events. For prediction of the expected proportion of super-spreading event, we produce a negative binomial distribution with dispersion parameter, *k*, and the mean number of secondary infections per index, *R*_*n*_ [4, 21].

### Identifying associations with index characteristics

The outcome variable was the number of secondary infections per index patient, which was found to be over-dispersed as described below. Therefore, we fitted a negative binomial regression model with both bivariate and multivariate regression with the MASS package [19] of the R environment for statistical computing [20]. The logarithmic scale coefficients were exponentiated to give ratios and are presented with their 95% confidence intervals (CI).

We evaluated the need for a negative binomial regression model (because of inequality of the conditional mean and conditional variance) with the likelihood ratio test. We also evaluated the predictive accuracy of the model with rootograms [22].

## Results

The Victorian TB program data had a total of 4190 confirmed TB index patients and 18,030 contacts within the period from 1 January 2005 to 31 December 2015. The mean age of index patients was 33.0 years and 54.9% were male. Among index patients, 1878 (44.8%) were extrapulmonary, while 1757 (42.0%) were pulmonary and 555 (13.2%) had both pulmonary plus other site involvement.

The average age of contacts was 28.4 years, 9276 (51.4%) were female and 8510 (47.2%) were male (with 244 (1.4%) contacts sex not stated). The majority of contacts (15,031; 83.4%) were contacts of pulmonary only TB patients, while (2988; 16.6%) were contacts of patients of TB at pulmonary plus other sites and only 11 contacts were identified from EPTB patients (although all EPTB were considered to have produced zero secondary infections and secondary cases). Henceforward we use the term “pulmonary” to refer to any patient with pulmonary involvement, i.e. both the “pulmonary only” and the “pulmonary plus other sites” categories. There were five categories for the types of contacts, 8059 close contacts, 5484 school contacts, 2366 work contacts, 1286 casual contacts and 824 contacts from other congregate setting such as hospitals, nursing homes, airlines and childcare facilities.

### Secondary infection distribution

A total of 15,522 of 18,019 contacts of PTB index patients were tested with TST. Based on our cut-off for diagnosing infection as those with a TST result of ≥10 mm as positive, 3213 of contacts were infected and 12,309 were not. Of 3213 infected contacts 2050 (63.8%) were close contacts. Of the 4190 index patients (1878 extrapulmonary and 2312 pulmonary) 3166 (75.6%) did not produce any secondary infection, with all extrapulmonary patients assumed not to have produced any secondary infections (Additional file 1). There were 26 cluster sizes of secondary infection, ranging from zero infections to 41 infections per index. The mean and variance of the number of secondary infections was 0.77 and 5.06 infections per index respectively. The median number of secondary infections per index patient was zero, the 95th centile was two and the 99th centile was 10 infections per index. By fitting the NBD to the observed distribution of secondary infection, we found evidence of over-dispersion (*k* = 0.16, 95%CI 0.14–0.17) for all types of contacts.

**Fig. 1:**
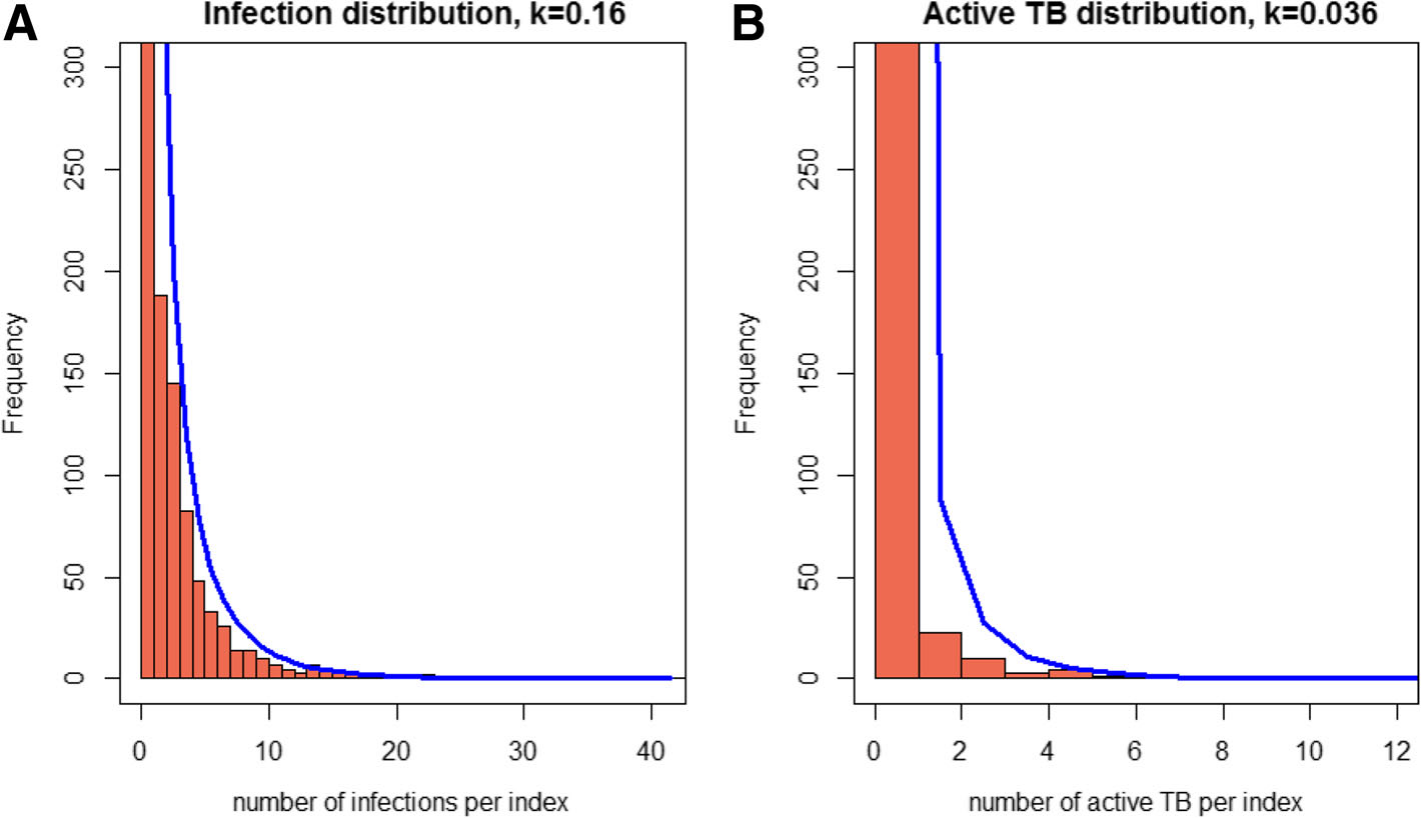
**a**. Distribution of number of secondary infections per index TB patient with negative binomial distribution fitted to count data in Victoria, for the period 2005–2015. The number of index patients with zero infections was 3166 (beyond limit of vertical axis). **b.** Distribution of secondary TB disease by index patients in Victoria, for the period 2005–2015, with negative binomial distribution fitted to count data. The number of index patients with zero secondary active TB was 4054 (beyond limit of vertical axis). Both panels include data for both PTB and EPTB index patients and all types of contacts

Restricting our analysis to index patients with pulmonary involvement only, the mean and variance of the number of infections per index was 1.4 and 8.3 infections per index, respectively with evidence of over-dispersion (*k*= 0.36, 95%CI 0.33–0.40) (Fig. 1a). In another restricted analysis of close contacts only, there were 2042 secondary infections, of which 771 (37.8%) were super-spreading events, with less dispersion compared to all contact types (*k*= 0.98, 95%CI 0.84–1.12).

### Super-spreading events

We constructed a Poisson distribution with the mean number of infections per index (i.e. 0.77) to establish a cut-off number of secondary infections per index for defining super-spreading events. The 99th centile was three infections per index. Therefore, we classified transmission events where index patients produced three or more secondary infections as super-spreading events. Accordingly, there were 414 (9.9% index patients) associated with super-spreading events, which accounted for a total of 2415 (75.2%) of the 3213 secondary infections. For predicting the number of super-spreading events in TB transmission, we estimated the expected proportion of index patients, with confidence intervals considering the dispersion parameter, *k* = 0.16 and the effective reproductive number,*R*_*n*_ = 0.77. With this approach, the expected proportion of TB super-spreading events was 9.8% (95% CI: 8.9–10.6%).

### Secondary active TB disease distribution

We further analysed infectiousness heterogeneity from the number of secondary active TB cases per index TB patient. There were 226 secondary active TB cases identified among 18,030 contacts. Among these secondary TB cases, approximately half (116; 51.3%) were the sole secondary case identified among the contacts of a specific index patient. Among 4190 index patients, only 137 (3.3%) were responsible for all 226 secondary TB cases. Two of the secondary cases were contacts of extrapulmonary index patients (maternal to foetal transmission in utero). The largest cluster of secondary TB disease was 12 cases. The distribution of secondary TB cases per index, was over dispersed with the dispersion parameter estimated at *k =* 0.036 (95%CI 0.025–0.046) (Fig. 1b).

### Contact distribution

We also investigated the individual variation among index patients with respect to the number of contacts identified to determine how the transmission heterogeneity could be related to contact patterns. There was evidence of heterogeneity (*k*= 0.38, 95%CI 0.36–0.41) for the distribution all contact types (Additional file 2). Similar but lesser heterogeneity was found (*k* = 0.63, 95%CI 0.59–0.68) after restricting our analysis to close contacts only (Additional file 3).

### Associations of index characteristics with number of infections

Because of the extent of heterogeneity described above, we fitted a negative binomial regression model for index patients with pulmonary involvement to determine the characteristics of index patients that were associated with the number of secondary infections. The index characteristics included were age, sex, site of disease, patient detection pathway, CXR result, method of diagnosis, whether the patient was new or relapse and number of contacts per index.

From this multivariate model, TB in pulmonary and additional sites (compared to pulmonary only) was independently associated with a 42% decrease in the number of secondary infections. Identification through contact tracing and the Australian post-migration follow up program (“health undertakings” [23]) (compared to clinical presentation) was associated with a lower number of secondary infections (71 and 46% respectively). Diagnosis by PCR, histology or clinical signs (compared to culture) was associated with a 70% decrease in secondary infections, while diagnosis by radiological techniques was associated with a 55% decrease. The number of contacts identified for each index patient showed a positive association with the number of secondary infections produced, with the identification of one additional contact associated with an increase in the number of secondary infections by 4 % (Table 1).

**Table 1:** Associations between number of secondary infections and patient characteristics among pulmonary TB index patients in Victoria, 2005–2015. Negative binomial regression model

Variable		Number of index patients	Crude RR (95%CI)	Adjusted RR (95%CI)
Age category (years)	0–14	140	0.43 (0.30–0.62)*	0.84 (0.56–1.26)
	15–24	486	1.60 (1.32–1.95)*	0.94 (0.80–1.11)
	25–44	890	Referent	
	45–64	378	0.77 (0.61–0.96)*	1.02 (0.85–1.22)
	65 and above	417	0.93 (0.75–1.15)	0.91 (0.77–1.09)
Sex	Female	954	Referent	
	Male	1357	0.95 (0.81–1.10)	0.93 (0.82–1.05)
Site of disease	Pulmonary	1757	Referent	
	Pulmonary plus additional sites	555	0.51 (0.43–0.62)*	0.58 (0.50–0.68)*
Patient detection pathway	Clinical Presentation	1839	Referent	
	Contact tracing	131	0.132 (0.08–0.21)*	0.29 (0.18–0.46)*
	Screening	24	0.49 (0.23–1.11)	0.64 (0.33–1.22)
	Health undertaking	317	0.37 (0.29–0.46)*	0.54 (0.44–0.67)*
CXR	Normal	31	Referent	
	Abnormal	879	1.79 (0.84–3.60)	1.18 (0.66–2.12)
	Unknown	1401	1.95 (0.91–3.91)	1.27 (0.71–2.26)
Diagnosis	Culture	1967	Referent	
	PCR/NAT/Ht/Cs	130	0.15 (0.09–0.23)*	0.30 (0.19–0.45)*
	Radiological	214	0.21 (0.15–0.29)*	0.45 (0.33–0.62)*
New or relapse	New case	2177	Referent	
	Relapse following full treatment	83	1.23 (0.84–1.86)	1.07 (0.78–1.47)
	Relapse following partial treatment	30	0.53 (0.26–1.14)	0.70 (0.38–1.25)
	Unknown	21	1.38(0.67–3.24)	1.02(0.56–1.91)
Number of contacts identified		18019^a^	1.06(1.05–1.06)*	1.04(1.04–1.05)*

*RR* rate ratio, *CI* confidence interval, *CXR* Chest X-Ray, *PCR/NAT/Ht/Cs* Polymerase chain reaction/nucleic acid test/histology/clinical signs, Health undertaking is post migration follow-up program

^a^=total number of contacts, * = statistically significant at *P* value < 0.05

The likelihood ratio test showed that assuming equality of the conditional mean and variance was not safe (*P*-value < 0.001), whereas model evaluation indicated that the negative binomial model fitted well to the observed data. Compared to the equivalent Poisson model, the negative binomial model was well-fitted and predicted counts well with minimal residuals (Additional file 4).

## Discussion

**Fig. 2:**
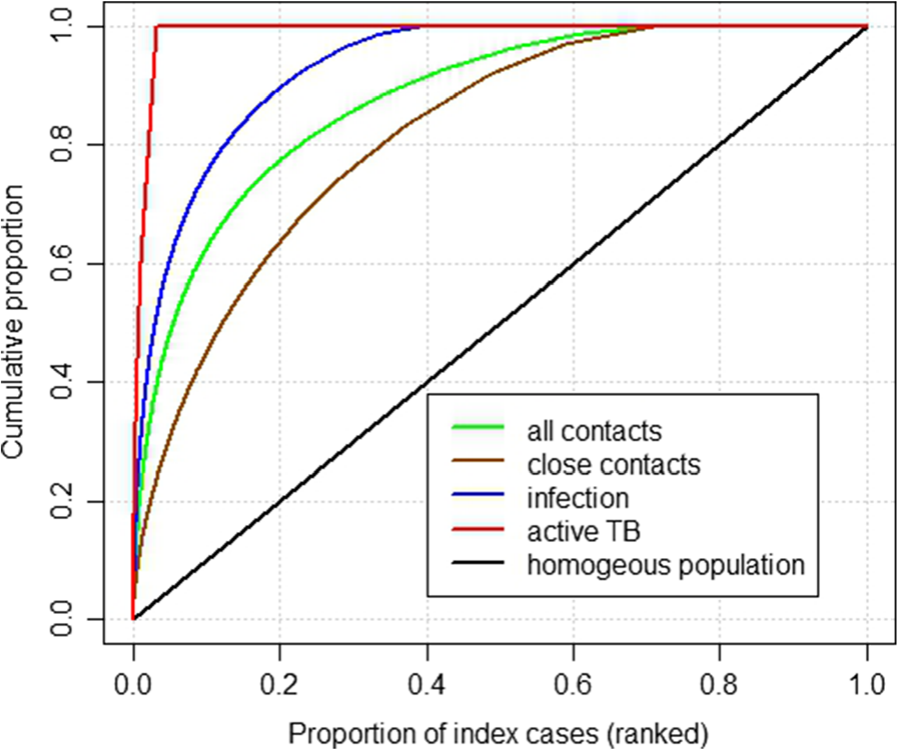
Proportion of all contacts, close contacts, proportion of secondary infections and secondary active TB disease due to a given proportion of infectious patients in Victoria, for the period 2005–2015

To our knowledge, this study is the first to use programmatic epidemiological observations to formally quantify *M. tb* transmission heterogeneity. We found evidence of super-spreading events as constituting the large majority of *M. tb* transmission events. We also demonstrated considerable variability between index TB patients in three important respects: in the number of contacts identified, the number of contacts infected and the number of cases of secondary active TB subsequently occurring. Therefore, assuming a homogeneous population of infective patients in TB transmission modelling may be highly unrealistic. From a programmatic viewpoint, although the effective reproduction number is less than one in our setting, significant transmission may still occur due to a very small number of super-spreading events (Fig. 2).

We found that *M. tb* transmission is heterogeneous, with the distribution of secondary infections per index varying by more than simple random variation between individuals. The level of over-dispersion was comparable with the estimate from a previous study that employed genotypic data (*k*=0.1) [10]. Although the distribution of secondarily infected contacts per index patient is probably a better marker for *M. tb* transmission than genotypic clusters, our approach may even underestimate heterogeneity (overestimate *k*) due to dilution of differences between patients from more homogeneously distributed distant past infection (i.e. prior to the index exposure identified). Our estimate of *k* was slightly higher (i.e. less heterogeneous) than an estimate for SARS transmission heterogeneity (*k*=0.1) [4], though our finding may under-estimate the true level of heterogeneity. The distribution of secondary cases was even more heterogeneous (*k*< 0.04) than secondary infections. This finding was also more heterogeneous compared to the previous TB estimate from genotypic data (k = 0.1) [10] and other infectious diseases such as SARS [4]. However, the effective use of preventive therapy in this setting and long incubation period could lead to an underestimate of the risk of active TB, as there is a risk of missing late reactivations of TB, although late reactivation are relatively rare in Victoria [24]. The effective use of preventive therapy could also overestimate the true level of heterogeneity in cases of secondary active TB, by differentially reducing the number of secondary cases produced by some index cases. The analyses of contacts demonstrated that these heterogeneities are not solely driven by heterogeneity in contact patterns. Therefore, we believe that the true value of the *M. tb* transmission dispersion parameter is likely to fall somewhere between the *k* estimate for secondary infections and secondary active TB distributions.

**Fig. 3:**
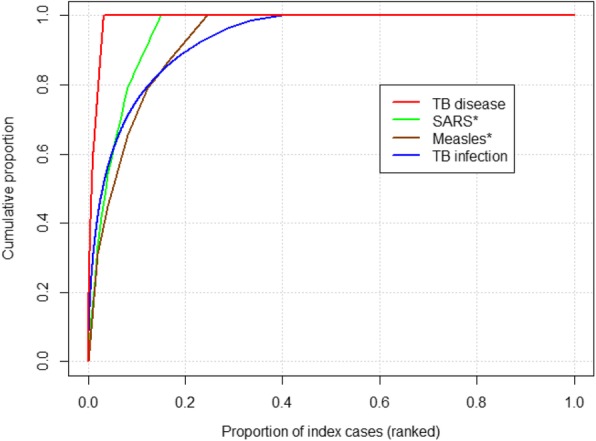
Comparison of proportion of secondary TB infections and secondary active TB disease with SARS and Measles due to a given proportion of index cases. *estimates taken from: Lloyd-Smith JO, Schreiber SJ, Kopp PE, Getz WM. Superspreading and the effect of individual variation on disease emergence. Nature. 2005;438(7066):355–9

Based on our definition [4], three-quarters of all secondary *M. tb* infections occurred as a result of super-spreading events. Although the average number of secondary infections per index (*R*_*n*_) was less than one (0.77), TB rates may not decline as expected in the population due to this high heterogeneity. Moreover, TB transmission goes well beyond the 20/80 rule-of-thumb for infectious disease transmission which states that 80% of transmission is due to only 20% of the population [25], since in our results 20% of index patients produce 90% of secondary infections (Fig. 3).

Our regression analysis identified several important associations between index patients’ characteristics and the number of secondary infections they produced, rather than considering just the proportion of contacts infected as typically done in previous studies [26, 27]. Compared to index patients with pulmonary TB only, index patients with TB involving pulmonary and non-pulmonary sites were less infectious. This may be explained by those with extrapulmonary TB but minor CXR abnormalities often being classified as “pulmonary plus other sites”, with these patients tending to have a low bacillary load and smear-negative pulmonary disease (given that it is well-established that pulmonary patients are more infectious than extrapulmonary who have virtually zero infectiousness [28, 29]). The method of patient identification was also an important predictor of infectiousness, with index patients found by clinical presentation being more infectious than those found through contact tracing or post-migration follow-up. This could be explained by the fact that those patients identified through the passive process of relying upon clinical presentation spend longer infectious, whereas those identified through the more active approaches of contact tracing and post-migration follow-up allows earlier identification and treatment. This explanation is consistent with delayed diagnosis and treatment being a major predictor of TB patients’ infectiousness [30–33]. Similarly, index patients diagnosed by culture produced a higher number of secondary infections, which is consistent with patients with culture-positive results having a higher bacillary load [34].

The most important limitation of our study is that some secondary infections might be the result of distant past infections, rather than relating to the contact episode. Our definition of super-spreading events is based on contact infection rather than active disease in contacts, since there is no standard definition in the case of TB and the difficulty in interpreting active disease in a setting of widespread use of preventive therapy. However, we argue that contact infection is the best available measure of true *M. tb* transmission in our setting and this definition could be used for future studies on the disease.

## Conclusions

We conclude that *M. tb* transmission is a highly heterogeneous process in our population and super-spreading events are a major driver of transmission. Therefore, it is essential to consider this heterogeneity when modelling TB transmission dynamics and considering control strategies. Future observational studies should characterise super-spreading in different epidemiological settings to further characterise this phenomenon.

## Additional files

Additional file 1: **Figure S1.** Schematic presentation of number of index TB patient and contacts in Victoria, for the period 2005–2015. (DOCX 52 kb)

Additional file 2: **Figure S2.** Distribution of number of contacts per index TB patient in Victoria, for the period 2005–2015. A. All contacts (with negative binomial distribution fitted to count data) strategies, the number of index patients with zero contacts was 639 (beyond limit of vertical axis). B. Subset of contacts (0–40 contacts per index only). (DOCX 34 kb)

Additional file 3: **Figure S3.** Distribution of number of Close contacts per index TB patient in Victoria, for the period 2005–2015. A. All Close contacts (with negative binomial distribution fitted to count data). B. Subset of close contacts (0–40 close contacts per index), the number of index patients with zero close contacts was 653 (beyond limit of vertical axis). (DOCX 34 kb)

Additional file 4: **Figure S4.** Hanging rootograms for a Poisson model (upper panel) and negative binomial model (lower panel) count data. (DOCX 37 kb)

## Abbreviations

BCG: Bacillus Calmette–Guérin
CI: Confidence Interval
CXR: Chest X-Ray
DHHS: Department of Health and Human Services
IGRA: Interferon Gamma Release Assay
LTBI: Latent Tuberculosis Infection
*M. tb*: Mycobacterium tuberculosis
PCR/NAT: Polymerase Chain Reaction/Nucleic Acid Test
RR: Rate Ratio
TB: Tuberculosis
TST: Tuberculin Skin Test
VTP: Victorian Tuberculosis Program
WHO: World Health Organization
