# Supplementary material for: The role of super-spreading events in Mycobacterium tuberculosis transmission: evidence from contact tracing
Source: BMC Infect Dis. 2019 Mar 12;19:244. doi: 10.1186/s12879-019-3870-1 (PMC6417041; doi:10.1186/s12879-019-3870-1)
Supplement: Supplementary file 1 — Figure S1. Schematic presentation of number of index TB patient and contacts in Victoria, for the period 2005–2015. (DOCX 52 kb) [file 12879_2019_3870_MOESM1_ESM.docx]

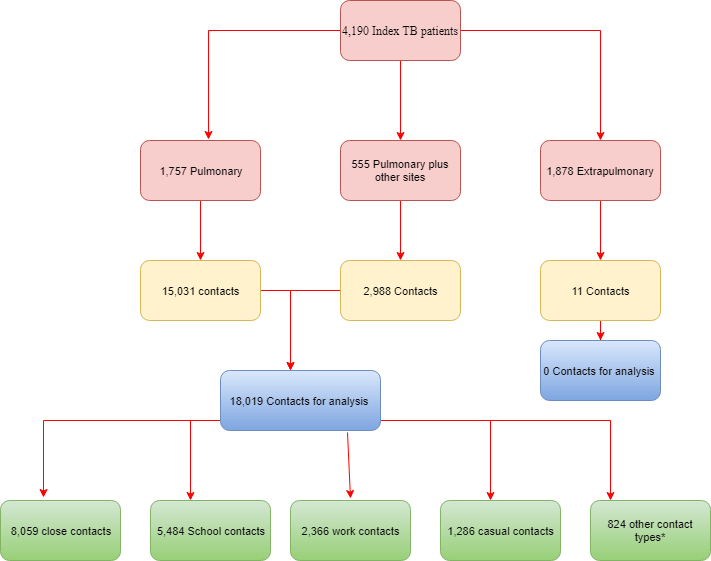


Other contact types* =includes contacts from hospitals, nursing homes, airlines and childcare facilities.

Figure S1: Schematic presentation of number of index TB patient and contacts in Victoria, for the period 2005-2015.
