# Supplementary material for: The role of super-spreading events in Mycobacterium tuberculosis transmission: evidence from contact tracing
Source: BMC Infect Dis. 2019 Mar 12;19:244. doi: 10.1186/s12879-019-3870-1 (PMC6417041; doi:10.1186/s12879-019-3870-1)
Supplement: Supplementary file 2 — Figure S2. Distribution of number of contacts per index TB patient in Victoria, for the period 2005–2015. A. All contacts (with negative binomial distribution fitted to count data) strategies, the number of index patients with zero contacts was 639 (beyond limit of vertical axis). B. Subset of contacts (0–40 contacts per index only). (DOCX 34 kb) [file 12879_2019_3870_MOESM2_ESM.docx]

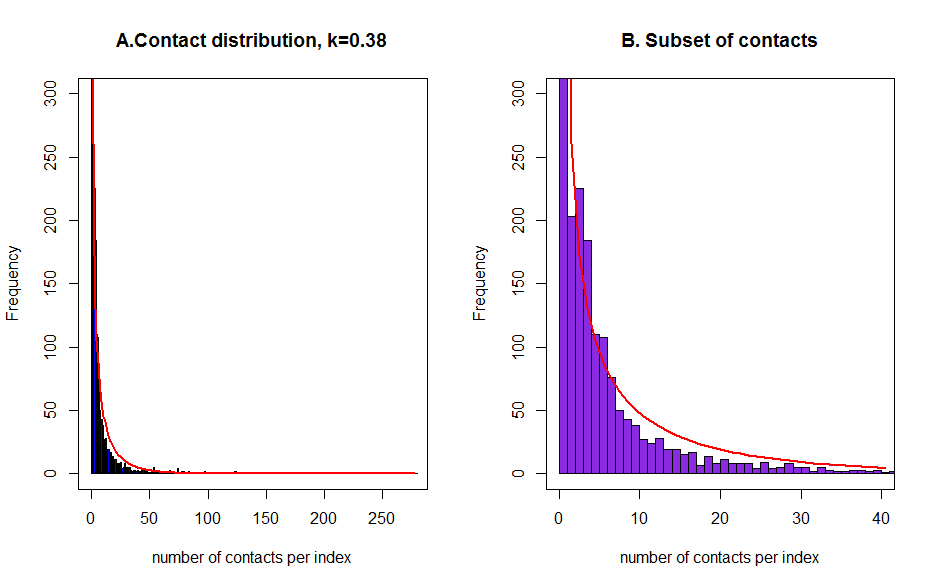


Figure S2: Distribution of number of contacts per index TB patient in Victoria, for the period 2005-2015. A. All contacts (with negative binomial distribution fitted to count data) strategies, the number of index patients with zero contacts was 639 (beyond limit of vertical axis). B. Subset of contacts (0-40 contacts per index only).
