# Supplementary material for: The role of super-spreading events in Mycobacterium tuberculosis transmission: evidence from contact tracing
Source: BMC Infect Dis. 2019 Mar 12;19:244. doi: 10.1186/s12879-019-3870-1 (PMC6417041; doi:10.1186/s12879-019-3870-1)
Supplement: Supplementary file 3 — Figure S3. Distribution of number of Close contacts per index TB patient in Victoria, for the period 2005–2015. A. All Close contacts (with negative binomial distribution fitted to count data). B. Subset of close contacts (0–40 close contacts per index), the number of index patients with zero close contacts was 653 (beyond limit of vertical axis). (DOCX 34 kb) [file 12879_2019_3870_MOESM3_ESM.docx]

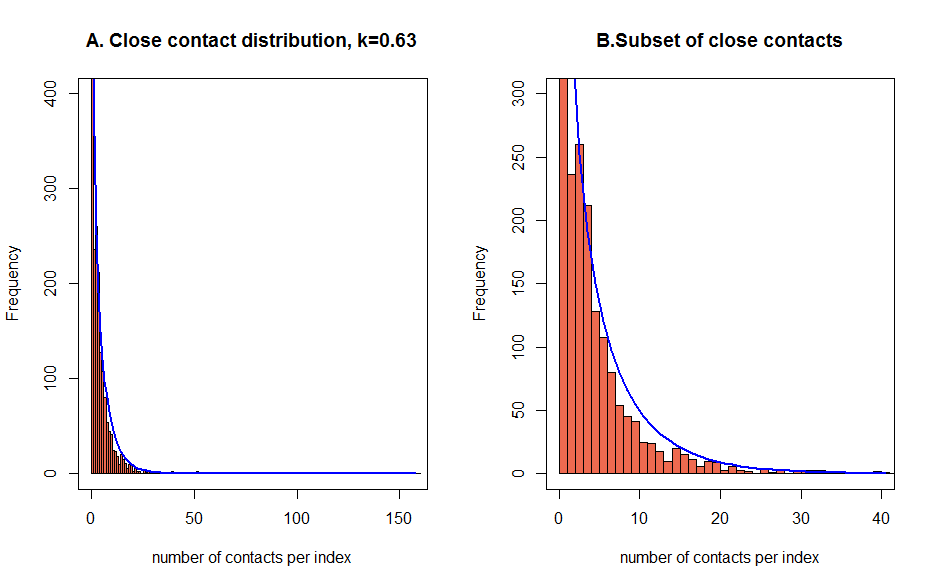


Figure S3: Distribution of number of Close contacts per index TB patient in Victoria, for the period 2005-2015. A. All Close contacts (with negative binomial distribution fitted to count data). B. Subset of close contacts (0-40 close contacts per index), the number of index patients with zero close contacts was 653 (beyond limit of vertical axis).
