# Supplementary material for: The role of super-spreading events in Mycobacterium tuberculosis transmission: evidence from contact tracing
Source: BMC Infect Dis. 2019 Mar 12;19:244. doi: 10.1186/s12879-019-3870-1 (PMC6417041; doi:10.1186/s12879-019-3870-1)
Supplement: Supplementary file 4 — Figure S4. Hanging rootograms for a Poisson model (upper panel) and negative binomial model (lower panel) count data. (DOCX 37 kb) [file 12879_2019_3870_MOESM4_ESM.docx]

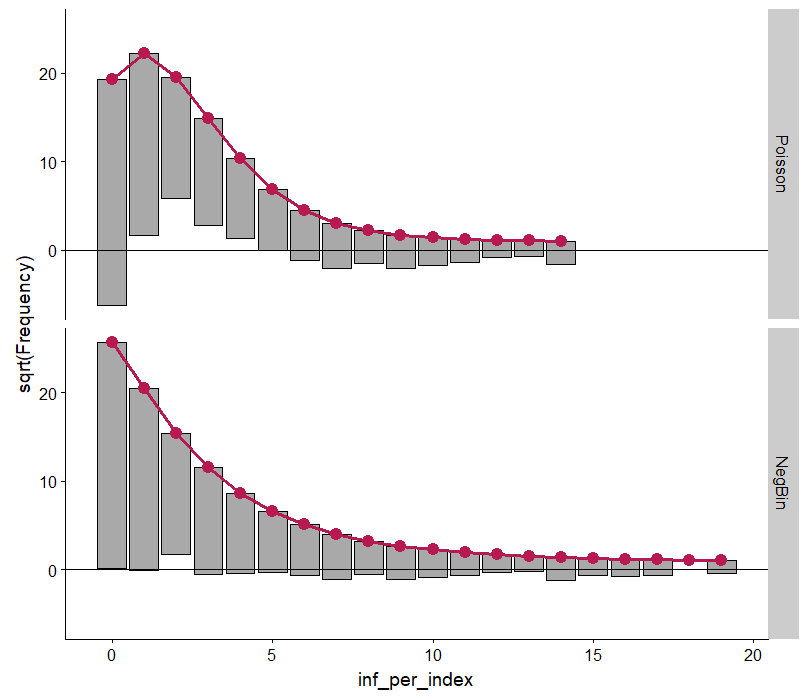


Figure S4: Hanging rootograms for a Poisson model (upper panel) and negative binomial model (lower panel) count data.

The figure shows the difference between predicted counts given the model (thick red line with dots) and observed counts (shown as bars hanging from the red dots). The x-axis is the outcome variable (number of secondary infections per index) while the y-axis is square root of the observed or expected count. The reference line is drawn at a height of zero, such that bars hanging below this reference line indicate under-prediction while bars hanging above zero indicate over-prediction. The Poisson model shows evidence of under-prediction for zero counts and most counts above five while the negative binomial model had less under prediction and predicted zero counts well. The Poisson model also over predicted the 1-4 counts, while in the negative binomial model over prediction was observed only for two counts. Therefore, the negative binomial model predicted counts well and considerably better than the Poisson model.
